# PAI-1 interaction with sortilin-related receptor 1 is required for lung fibrosis

**DOI:** 10.1172/jci.insight.186131

**Published:** 2025-04-29

**Authors:** Thomas H. Sisson, John J. Osterholzer, Lisa Leung, Venkatesha Basrur, Alexey Nesvizhskii, Natalya Subbotina, Mark Warnock, Daniel Torrente, Ammara Q. Virk, Sergey S. Gutor, Jeffrey C. Horowitz, Mary Migliorini, Dudley K. Strickland, Kevin K. Kim, Steven K. Huang, Daniel A. Lawrence

**Affiliations:** 1 Division of Pulmonary and Critical Care Medicine, Department of Internal Medicine, University of Michigan Medical School, Ann Arbor, Michigan, USA.; 2 Research Service, Ann Arbor VA Health System, Department of Veterans Affairs Health System, Ann Arbor, Michigan, USA.; 3 Department of Molecular Integrative Physiology,; 4 Department of Pathology-Proteomic Resource Facility,; 5 Department of Computational Medicine and Bioinformatics, and; 6 Division of Cardiovascular Medicine, Department of Internal Medicine, University of Michigan Medical School, Ann Arbor, Michigan, USA.; 7 Division of Pulmonary and Critical Care Medicine, Department of Internal Medicine, Ohio State University, Columbus, Ohio, USA.; 8 Center for Vascular and Inflammatory Disease and Departments of Surgery and Physiology, University of Maryland School of Medicine, Baltimore Maryland, USA.; 9 Department of Molecular and Integrative Physiology, University of Michigan, Ann Arbor, Michigan, USA.

**Keywords:** Aging, Pulmonology, Fibrosis, Plasmin, Protein traffic

## Abstract

Mutation studies of plasminogen activator inhibitor 1 (PAI-1) have previously implied that PAI-1 promotes lung fibrosis via a vitronectin-dependent (VTN-dependent) mechanism. In the present study, employing 2 distinct murine fibrosis models and VTN-deficient mice, we found that VTN is not required for PAI-1 to drive lung scarring. This result suggested the existence of a profibrotic interaction involving the VTN-binding site on PAI-1 with an unidentified ligand. Using an unbiased proteomic approach, we identified sortilin-related receptor 1 (SorLA) as the most highly enriched PAI-1 binding partner in the fibrosing lung. Investigating the role of SorLA in pulmonary fibrosis demonstrated that deficiency of this protein protected against lung scarring in a murine model. We further found that SorLA is required for PAI-1 to promote scarring in mice, that both SorLA and PAI-1 protein levels are increased in human idiopathic pulmonary fibrosis (IPF) explants, and that these proteins are associated in IPF tissue. Finally, confocal microscopy showed that expression of SorLA in CHO cells increased cellular uptake of PAI-1, and these proteins colocalized in the cytoplasm. Together, these data elucidate a mechanism by which the potent profibrotic mediator PAI-1 drives lung fibrosis and implicate SorLA as a potential therapeutic target in IPF treatment.

## Abstract 2

We uncovered an interaction between PAI-1 and SorlA in the context of pulmonary fibrosis, and we determined that SorlA is required for lung scarring.

## Introduction

Diseases resulting in lung fibrosis remain challenging clinical problems. Idiopathic pulmonary fibrosis (IPF), the prototypical scarring disorder, is associated with significant morbidity and mortality (1). Two FDA-approved drugs are now available for patients with IPF, but neither halts disease progression (1). Therefore, more effective treatments are needed to improve the outcome of IPF and other fibrotic diseases. Plasminogen activator inhibitor 1 (PAI-1) represents an attractive therapeutic target for lung fibrosis based on data from several preclinical models employed in multiple laboratories (2–6). Specifically, transgenic deletion of PAI-1 attenuates fibrosis in complementary models (2, 5, 6), whereas constitutive PAI-1 overexpression exacerbates scarring following bleomycin-induced injury (2). Our studies confirm that PAI-1 promotes fibrosis not by modifying the early lung injury, but rather through an activity exerted during the fibrotic phase of these models (7). Despite the substantial evidence establishing a causal role for PAI-1, the precise mechanism by which PAI-1 promotes lung fibrosis remains unknown.

PAI-1 is a multifunctional protein with inhibitory activity against endogenous plasminogen activators. By reducing plasmin activity, PAI-1 favors the persistence of fibrin in areas of tissue injury (5, 8). PAI-1 also binds to the somatomedin B (SMB) domain of vitronectin (VTN), a provisional matrix molecule. Binding to VTN enhances PAI-1 stability and disrupts cellular adherence to VTN (9–13). In prior studies using transgenic mice and PAI-1 proteins that lack specific functions, we determined that amino acids in PAI-1 necessary for VTN binding are critical for the profibrotic activity of PAI-1 (7), and we hypothesized that an interaction between PAI-1 and VTN was necessary for PAI-1–dependent fibrogenesis. We speculated that PAI-1 binding to VTN might disrupt the protective effects of this provisional matrix molecule on alveolar epithelial cell survival and wound repair, processes thought to counteract fibrosis (14, 15).

In the present study, we interrogated the importance of VTN to the profibrotic activity of PAI-1 using 2 distinct lung fibrosis models. Surprisingly, we found that PAI-1 promotes lung scarring in the presence or absence of VTN. This observation prompted an unbiased proteomic analysis to identify potentially important PAI-1 binding partners in the injured lung that might regulate its profibrotic activity. We identified sortilin-related receptor 1 (SorLA) as the most highly enriched PAI-1 interactor. SorLA is a multidomain, mosaic receptor involved in internalizing and sorting cargo proteins (16, 17). Prior studies identified roles for SorLA in human disease, but to our knowledge, this protein has not been studied in the context of pulmonary fibrosis (18). After identifying an interaction between PAI-1 and SorLA, we established that the binding site on PAI-1 for SorLA overlaps with its VTN binding site. Furthermore, we detected increased PAI-1 and SorLA expression in lung tissue explants from patients with IPF. Using SorLA-heterozygous and -deficient mice, we established a gene-dose effect in the development of lung fibrosis. Importantly, we confirmed that SorLA expression is necessary for PAI-1 to exert its profibrotic activity and that SorLA and PAI-1 colocalize within cells. Collectively, our data identify SorLA as a profibrotic cofactor for PAI-1 in the injured lung and implicate SorLA in the development of pulmonary fibrosis.

## Results

### The profibrotic effects of PAI-1 are independent of VTN.

**Figure 1:**
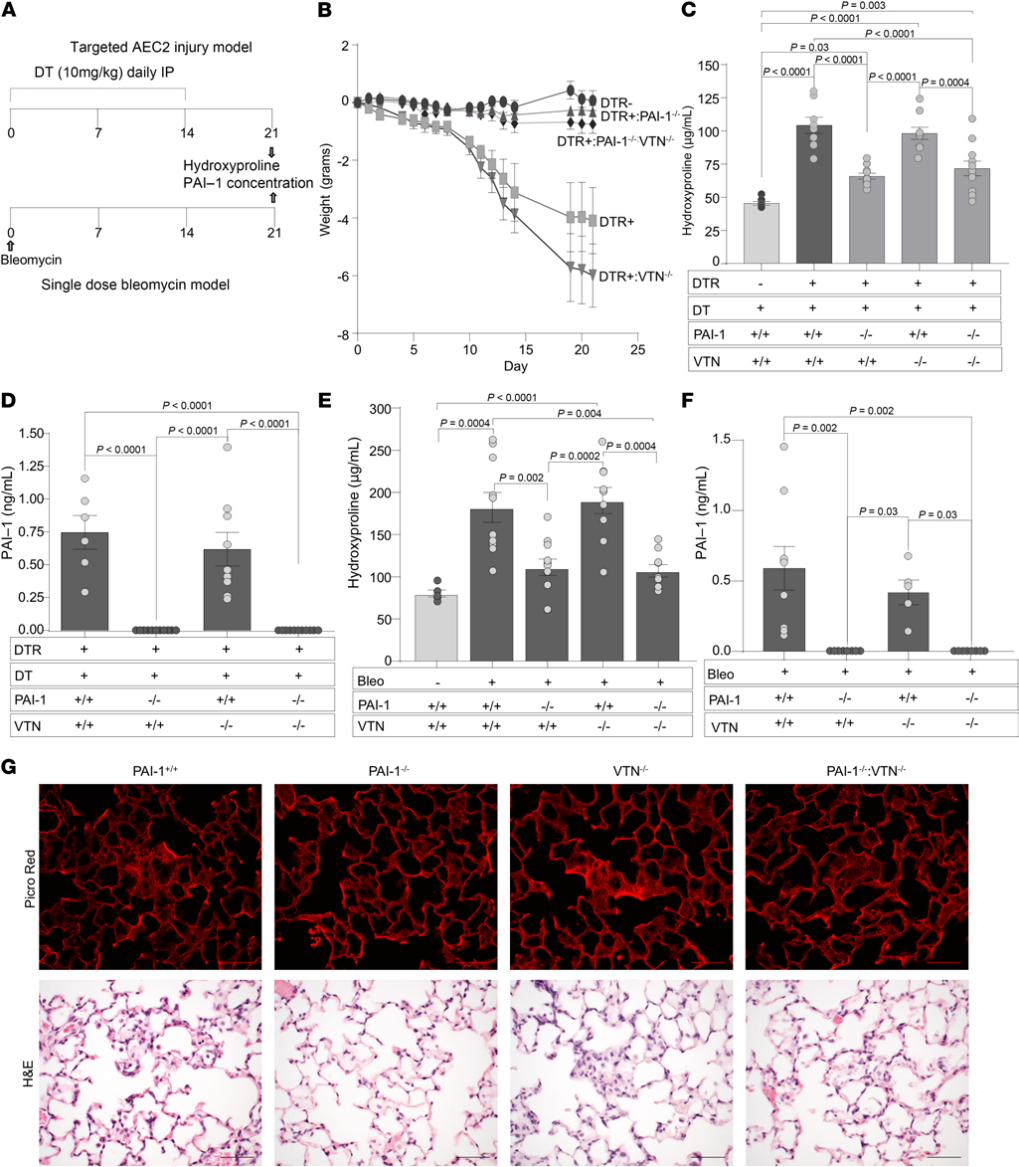
The profibrotic effects of PAI-1 following AEC2 injury are independent of VTN. (**A**) Schematics of murine fibrosis models. In the targeted AEC2 injury model, DT (12.5 μg/kg) was administered for 14 days to (i) *DTR+* mice, (ii) *DTR+*:*PAI-1^–/–^* mice, (iii) *DTR+*:*VTN^–/–^* mice, and (iv) *DTR+*:*PAI-1^–/–^*:*VTN^–/–^* mice. A control cohort of WT (*DTR*–) mice treated with DT was also included in the study protocol. In the single-dose bleomycin model, bleomycin was administered (2.5 U/kg in 50 μL by the oropharyngeal route) on day 0 to (i) *WT* mice, (ii) *PAI-1^–/–^* mice, (iii) *VTN^–/–^* mice, and (iv) double-knockout *PAI-1^–/–^*:*VTN^–/–^* mice. A group of uninjured WT mice were included as a negative control. (**B**) In the targeted AEC2 injury model, mice were weighed at regular intervals. (**C** and **E**) Lungs were harvested on day 21 (D21) and analyzed for hydroxyproline content. (**D** and **F**) BAL samples were obtained on D21 and assayed for total PAI-1 levels. Results in **B**–**F** are reported as the mean concentration ± SEM. *n* = 6–7 (**B**), *n* = 7–11 (**C**), *n* = 5–11 (**D**), *n* = 5–11 (**E**), and *n* = 5–8 (**F**). Representative data are displayed from 1 of 3 (**A** and **B**) and 1 of 2 (**D**) experiments. Significant *P* values are shown from 2-way ANOVA with Tukey’s multiple-comparison test. (**G**) H&E- and Picrosirius red–stained D21 lung sections from a representative animal in the targeted AEC2 injury model. Scale bars: 180 μm.

Previously, we found that a PAI-1 variant with intact VTN binding but no protease inhibitory activity could fully restore lung fibrosis in bleomycin-injured *PAI-1^–/–^* mice (7). This result led us to hypothesize that VTN might play an antifibrotic role following lung injury, and that PAI-1 binding to VTN might abolish this protective effect. To investigate this hypothesis, we employed 2 complementary murine models of pulmonary fibrosis as recommended by the 2017 ATS Workshop Report (19). One model involves selective type 2 alveolar epithelial cell (AEC2) injury via repetitive doses of diphtheria toxin (DT) administered to mice expressing the DT receptor (DTR) under the transcriptional control of the surfactant protein C (SPC) promoter (*DTR+*; Figure 1A). The second model involves a single, weight-based dose of intrapulmonary bleomycin administered on day 0 with lung fibrosis assessed on day 21 (Figure 1A). In both models, as recommended by the 2017 ATS Workshop (19), we used hydroxyproline, a biochemical measure of collagen, to quantify the severity of lung fibrosis, and we analyzed histopathology sections to evaluate the pattern of scarring. We employed both models to interrogate the following groups of mice: (a) WT, (b) *PAI-1^–/–^*, (c) *VTN^–/–^*, and (d) *PAI-1^–/–^*:*VTN^–/–^*. In both models, untreated WT mice were used as negative controls because we found no difference in baseline lung collagen content between WT, *VTN^–/–^*, and *PAI-1^–/–^* genotypes (Supplemental Figure 1; supplemental material available online with this article; https://doi.org/10.1172/jci.insight.186131DS1). In addition, in the targeted AEC2 injury model, WT mice treated with DT were included to assess nonspecific effects of DT.

We found that DT injury to both *DTR+*:WT and *DTR+*:*VTN^–/–^* mice resulted in prominent weight loss relative to the DT-exposed *DTR–* group whose weight remained stable over the 21-day protocol (Figure 1B). In contrast, the *DTR+*:*PAI-1^–/–^* and the *DTR+*:*PAI-1^–/–^*:*VTN^–/–^* mice exhibited less weight loss in response to injury. Our quantification of lung fibrosis revealed that the 2 groups with WT PAI-1 expression (*DTR+*:WT and *DTR+*:*VTN^–/–^*) developed statistically significantly increased lung collagen content in response to DT (relative to the negative control group), while the 2 groups deficient in PAI-1 expression (*DTR+*:*PAI-1^–/–^* and *DTR+*:*PAI-1^–/–^*:*VTN^–/–^*) were protected (Figure 1C). Day 21 histologic evaluation revealed patchy areas of interstitial thickening, increased cellularity, and enhanced Picrosirius red staining in the *DTR+*:WT and *DTR+*:*VTN^–/–^* mice as compared with the PAI-1–deficient groups (Figure 1G).

To assess for the possibility that VTN deficiency might affect PAI-1 levels in this model and thereby influence fibrogenesis, we measured PAI-1 in BAL samples obtained from each cohort of mice. Our results demonstrated, as expected, no detectable PAI-1 in the *PAI-1^–/–^* groups. We also observed no difference in PAI-1 concentrations between the DT-injured *DTR+*:WT and *DTR+*:*VTN^–/–^* mice (Figure 1D).

In the single-dose intrapulmonary bleomycin injury model, we found congruent results with statistically significant increases in lung collagen content in injured WT and *VTN^–/–^* mice (relative to uninjured WT mice; Figure 1E), and attenuated fibrosis in the PAI-1–deficient groups irrespective of whether VTN was present or absent. Furthermore, the presence or absence of VTN did not affect PAI-1 levels in the BAL fluid of mice following bleomycin-induced injury (Figure 1F).

### Reconstitution of PAI-1–deficient mice with recombinant PAI-1 restores pulmonary fibrosis in the absence of VTN.

**Figure 2:**
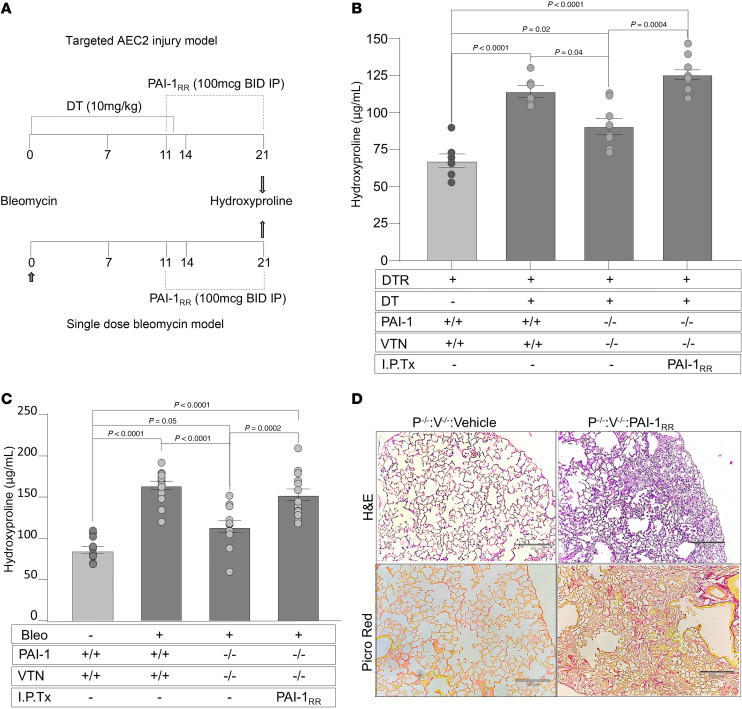
Reconstitution of *PAI-1^–/–^*:*VTN^–/–^* mice with PAI-1_RR_ restores pulmonary fibrosis in a VTN-independent manner. (**A**) Schematics of murine fibrosis models. In the targeted AEC2 injury model, DT (10.0 μg/kg) was administered for 14 days to *DTR+* and *DTR+*:*PAI-1^–/–^*:*VTN^–/–^* mice. On day 11 (D11), DT-injured *DTR+*:*PAI-1^–/–^*:*VTN^–/–^* mice received either i.p. recombinant PAI-1_RR_ (deficient antiprotease activity but intact VTN binding) at 100 μg twice daily or an equivalent volume of PBS. Control cohorts included DT-injured *DTR*+ mice and uninjured *DTR*+ mice (both without PAI-1_RR_ reconstitution). In the single-dose bleomycin model, bleomycin was administered (2.5 U/kg) to double-knockout *PAI-1^–/–^*:*VTN^–/–^* mice. Beginning on D11, *PAI-1^–/–^*:*VTN^–/–^* mice were treated with either recombinant PAI-1_RR_ or PBS. Control cohorts of mice receiving PBS in place of PAI-1_RR_ included bleomycin-injured and uninjured WT mice. (**B** and **C**) Lungs were harvested on D21 and analyzed for hydroxyproline content. (**D**) Harvested lungs were inflating fixed, sectioned, and stained with H&E (top panels) and Picrosirius red (bottom panels). Scale bars: 180 μm. Results in **B** and **C** are reported as the mean concentration ± SEM. *n* = 7–10 (**B**), *n* = 11–15 (**C**). Representative data are displayed from 1 of 3 (**B**) and 1 of 2 experiments (**C**). Significant *P* values are shown for comparisons performed using 2-way ANOVA with Tukey’s multiple-comparison test.

To further rule out a contribution of VTN in mediating the profibrotic activity of PAI-1, we employed a second experimental approach. We treated injured *PAI-1^–/–^*:*VTN^–/–^* mice with either PBS or PAI-1_RR_, a PAI-1 variant with intact VTN-binding activity but no plasminogen activator inhibitory activity (20). PAI-1_RR_ was administered during the fibrotic phase from day 11 through day 21 in both models (Figure 2A). This protocol was informed by previously published data in which we showed that reconstituting bleomycin-injured *PAI-1^–/–^* animals with PAI-1_RR_ restored lung fibrosis to a level comparable to bleomycin-injured WT animals (7). We quantified lung fibrosis in each model with hydroxyproline and assessed the pattern of fibrosis via lung histology. For the targeted AEC2 injury model, PBS-treated *DTR+*:WT mice served as a negative control group, while DT-treated *DTR+*:WT mice were included as a positive control. For the single-dose intrapulmonary bleomycin injury model, uninjured WT mice served as a negative control while bleomycin-injured WT animals were included as a positive control.

In the AEC2 injury model and consistent with data in Figure 1C, the DT-injured *DTR+*:*PAI-1^–/–^*:*VTN^–/–^* group developed attenuated fibrosis when compared with the DT-injured *DTR+*:WT animals (Figure 2B). Reconstitution of the DT-injured *DTR+*:*PAI-1^–/–^*:*VTN^–/–^* group with PAI-1_RR_ resulted in statistically significant lung collagen accumulation that was comparable to, and not statistically different from, the DT-injured *DTR+*:WT group.

In the single-dose intrapulmonary bleomycin model, we observed similar findings in that the *PAI-1^–/–^*:*VTN^–/–^* group accumulated less hydroxyproline following injury when compared with the bleomycin-injured WT group. Reconstitution of *PAI-1^–/–^*:*VTN^–/–^* mice with PAI-1_RR_, as in the AEC2 injury model, worsened lung fibrosis as assessed by hydroxyproline (Figure 2C) and lung histologic analysis that revealed more extensive fibrotic lesions in H&E- and Picrosirius red–stained sections (Figure 2D).

Together, data from these complementary models demonstrate that the presence of PAI-1 during the fibrotic phase of lung injury is critical for the development of lung fibrosis whether VTN is present or not (Figures 1 and 2). These observations support the conclusion that the profibrotic function of PAI-1 is acting independently of VTN and suggest that the residues required for VTN binding in PAI-1 interact with another target to exacerbate fibrosis.

### Identification of SorLA as a PAI-1 binding partner in pulmonary fibrosis.

After finding that VTN is not required for the profibrotic activity of PAI-1, we sought to identify additional PAI-1 interactors in the injured lung. To accomplish this goal, we incubated homogenized lung tissue from *PAI-1^–/–^* mice (collected on day 10 after bleomycin administration) with streptavidin beads coated with biotin-tagged PAI-1. To a subset of samples, a reversible cross-linker (DSP) was added to enhance the detection of low-affinity interactions. We then analyzed the abundance of proteins (spectral counts) bound to the PAI-1–coated beads relative to control beads via mass spectrometry. Spectral counts for each identified protein were represented as the log_2_(fold change) (log_2_FC) between PAI-1 and control samples with (FC+) and without (FC–) cross-linker. Finally, we calculated an average of FC+ and FC– for each protein. With this approach, we identified SorLA as the most highly enriched PAI-1 binding partner, and as a validation of our technique, we also detected both tissue plasminogen activator (tPA) and VTN within the list of the top 25 most enriched proteins (Table 1).

**Figure 3:**
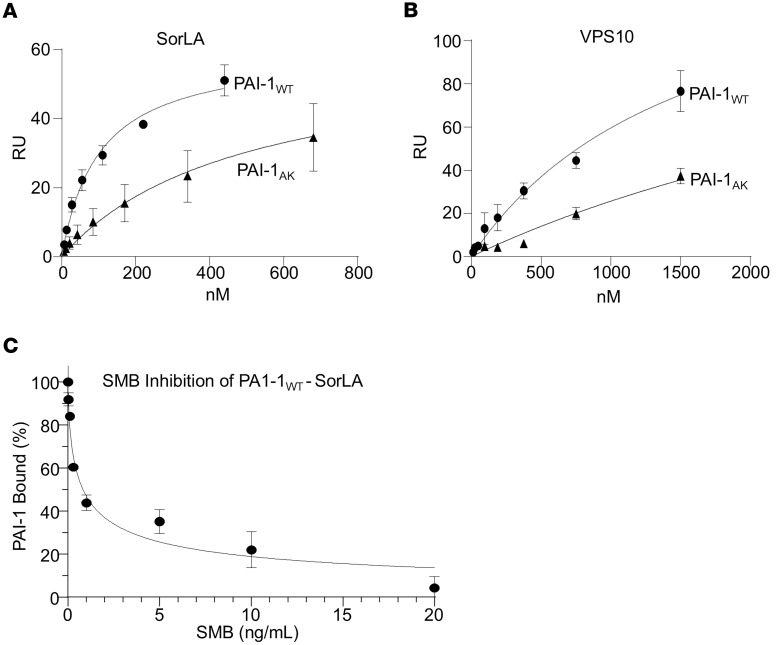
PAI-1 binds to SorLA as revealed by SPR analysis. Increasing concentrations of PAI-1_WT_ or PAI-1_AK_ were flowed over SPR flow cells coupled with soluble SorLA (**A**) or the VSP10 domain (**B**). Data were analyzed by equilibrium analysis by fitting the association data to a first-order process to determine *R_eq_*. The data were normalized to *R_max_* and plotted as *R_eq_*/*R_max_* versus PAI-1 concentrations. Data were fit to a single binding site by nonlinear regression analysis. (**C**) Inhibition of PAI-1 (250 nM) binding to SorLA in the presence of increasing concentrations of somatomedin B (SMB) domain of VTN.

To verify our finding that PAI-1 binds SorLA, we employed surface plasmon resonance (SPR). SorLA is a multidomain protein that contains a region homologous to the LDL receptor (LDLR) family of proteins, and prior studies demonstrate binding of PAI-1 to LDLR family members, including lipoprotein receptor–related protein 1 (LRP1) and SorLA (21). SorLA also contains a vacuolar protein sorting 10 domain (VPS10), which has been shown to traffic proteins between different cytoplasmic compartments. In this experiment, we analyzed binding of PAI-1 to the isolated VPS10 domain and to full-length SorLA by immobilizing both proteins on an SPR chip. Solutions containing increasing concentrations of PAI-1_WT_ and PAI-1_AK_ — a mutant with intact protease inhibitory activity, no detectable affinity for VTN (22), and attenuated profibrotic activity (7) — were flowed over the soluble forms of full-length SorLA and the isolated SorLA VPS10 domain to assess binding. As represented in Figure 3A, PAI-1_WT_ bound 5-fold more avidly to full-length SorLA than PAI-1_AK_, with respective *K_D_* values of 126 ± 47 nM versus 540 ± 95 nM (Table 2). Both PAI-1_WT_ and PAI-1_AK_ also bound to the VPS10 domain of SorLA, an interaction that, to our knowledge, has not been previously reported (Figure 3B and Table 2). Like full-length SorLA, PAI-1_WT_ bound more avidly to the VPS10 domain of SorLA than PAI-1_AK_. To confirm overlap between the PAI-1 binding site on VTN and full-length SorLA, we assessed the binding of PAI-1_WT_ to SorLA in the presence of increasing concentrations of the SMB domain of VTN. We found that SMB inhibits PAI-1_WT_ binding to SorLA with an IC_50_ of 464 nM (Figure 3C). This observation supports the conclusion that the binding sites on PAI-1 for VTN and SorLA overlap.

**Figure 4:**
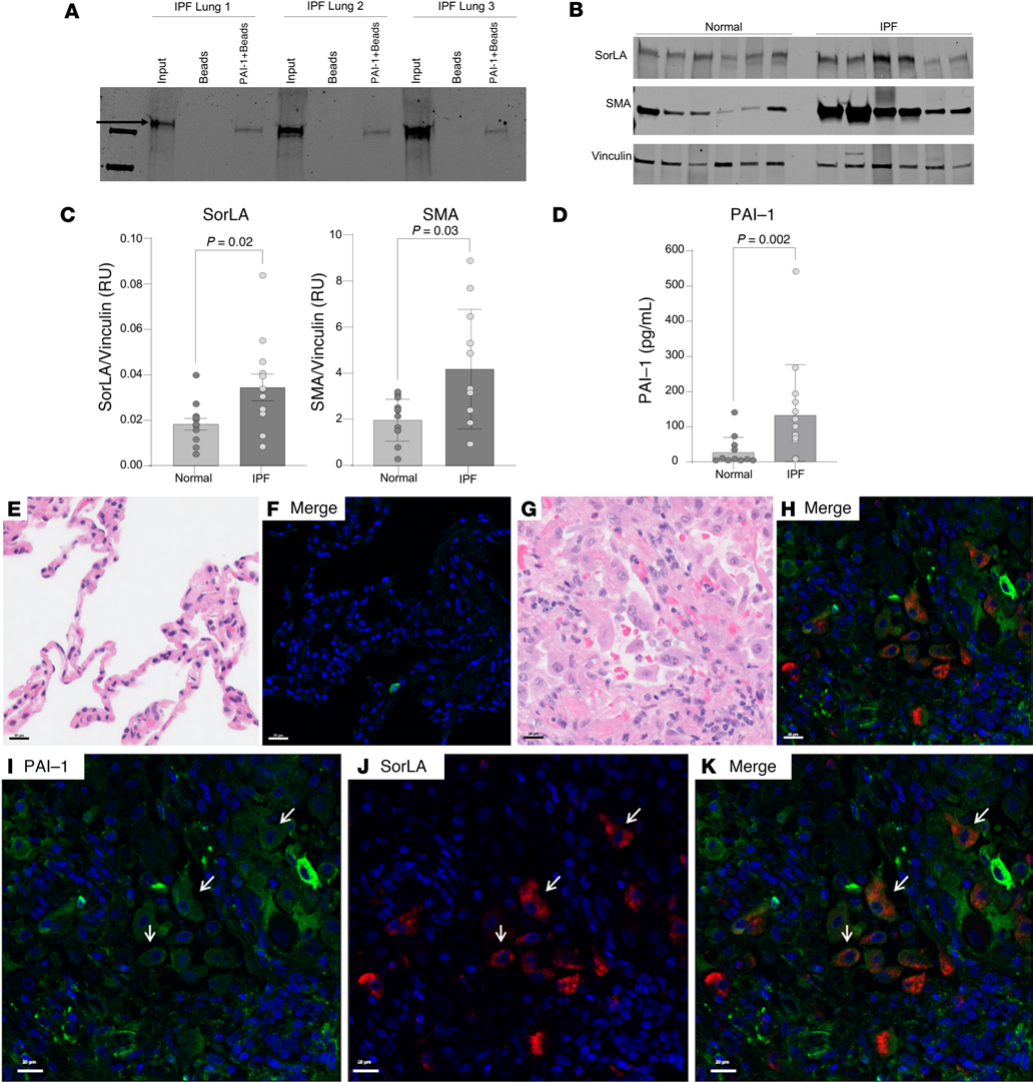
PAI-1_WT_ binds to SorLA in lung tissue homogenates from patients with IPF. (**A**) Fibrotic lung tissue obtained from explants at the time of transplant were homogenized in binding buffer. Each sample (200 μg) was incubated with either uncoated magnetic streptavidin-Sepharose beads or beads coated with biotin-tagged PAI-1_WT_. Beads were collected, washed, and proteins were eluted with SDS loading buffer. The initial homogenate (input) and the eluted proteins (Beads, PAI-1-Beads) were separated by SDS-PAGE, blotted, and stained with an anti-SorLA antibody. Data are displayed as a representative gel. (**B**) Equal quantities of protein from homogenized IPF or normal control lung tissue were separated by SDS-PAGE and analyzed by Western blotting for SorLA and αSMA (normalized to vinculin, *n* = 13). (**C**) Quantification of SorLA and αSMA in fibrotic tissue. (**D**) Active PAI-1 levels measured by ELISA (normalized to total lung protein concentration, *n* = 13). (**E**–**K**) Immunofluorescent costaining of PAI-1 (Akoya Opal 520, green) and SorLA (Akoya Opal 690, red) in normal (**E** and **F**) and IPF (**G**–**K**) human lung tissue sections. (**I**–**K**) Enlargement of panel **H** with (**I**) DAPI and PAI-1, (**J**) DAPI and SorLA, and (**K**) merged. Scale bars: 20 μm. Data are represented as mean ± SEM. Significant *P* values are shown for comparisons performed using a parametric 2-tailed *t* test (Panel **C**, **D**) or Mann–Whitney *U* nonparametric 2-tailed *t* test.

To confirm binding between PAI-1 and SorLA in human fibrotic lung tissue, we added PAI-1–coated streptavidin beads to homogenized explanted lungs obtained from patients with end-stage fibrotic disease. The captured proteins were eluted, separated by gel electrophoresis, and immunoblotted for SorLA. With this approach, we found that PAI-1 binds to SorLA in fibrotic human lung tissue (Figure 4A). We also found that SorLA, along with α-smooth muscle actin (αSMA) and PAI-1, is upregulated in lung tissue from patients with end-stage lung fibrosis (predominantly IPF) versus healthy controls (Figure 4, B–D). Of note, we identified a statistically significant correlation between SorLA and αSMA in the human lung samples (Supplemental Figure 2). To determine whether PAI-1 and SorLA are associated in fibrotic human lung tissue, immunofluorescent costaining of PAI-1 and SorLA was performed in both normal human lung and end-stage IPF lung sections. These data demonstrated that, unlike normal lung where both PAI-1 and SorLA were only sporadically observed and were never colocalized (Figure 4, E and F), in fibrotic lung tissue, PAI-1 and SorLA were both expressed in individual cells (Figure 4, G–K). There were more cells expressing PAI-1 than SorLA but most cells expressing SorLA were also PAI-1 positive, suggesting a possible direct interaction of these proteins in fibrotic tissue. Taken together, these results indicate that both PAI-1 and SorLA are upregulated in human fibrotic lung disease, and that PAI-1 can be colocalized in cells expressing SorLA.

### Deficiency of SorLA protects against lung fibrosis.

**Figure 5:**
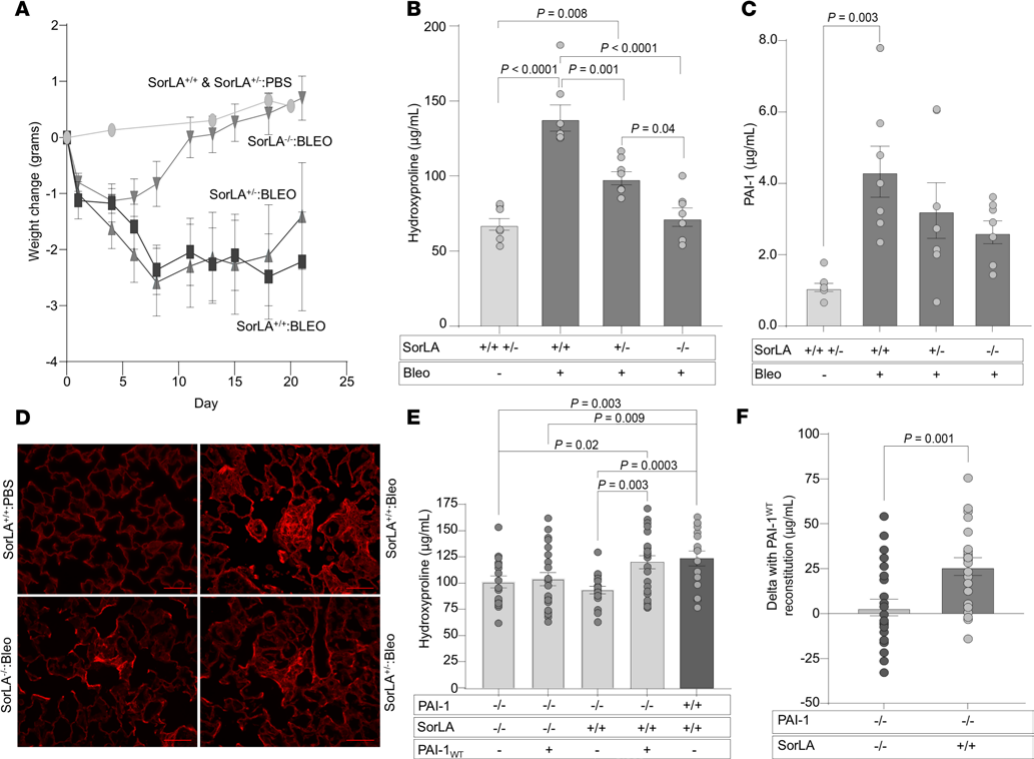
SorLA-deficient and SorLA-heterozygous mice are protected from fibrosis following lung injury. (**A**–**C**) Single-dose bleomycin (2.5 U/kg in 50 μL) was administered on day 0 (D0) to the following littermate cohorts: (i) *SorLA^+/+^*, (ii) *SorLA^+/–^*, and (iii) *SorLA^–/–^* mice. Control littermates (mix of *SorLA^+/+^*, *SorLA^+/–^*, *SorLA^–/–^*) were uninjured and served to establish baseline lung collagen content. (**A**) Mice were weighed at regular intervals (*n* = 7–8/group) and (**B** and **C**) lungs and BAL were collected on D21 for hydroxyproline analysis and measurement of PAI-1 concentration (*n* = 7–8). Representative data from 1 of 3 experiments are shown (**B**), and results are reported as the mean ± SEM. *P* values are displayed for comparisons performed using 2-way ANOVA with Tukey’s post hoc multiple-comparison test. (**D**) Lung sections obtained on D21 were stained with H&E (left panel) and Picrosirius red (right panel) and representative images are shown. Scale bars: 180 μm. Single-dose bleomycin was administered (2.5 U/kg in 50 μL) on D0 to the following littermate cohorts: (i) *PAI-1^–/–^*:*SorLA^+/+^* and (ii) *PAI-1^–/–^*:*SorLA^–/–^*. On D11, subsets of mice from each genotype were administered either i.p. recombinant PAI-1_WT_ at 100 μg twice daily or an equivalent volume of PBS. On D21, lungs were analyzed for hydroxyproline content. (**E**) Mean hydroxyproline values in each group (*n* = 17–24/group). (**F**) Delta in hydroxyproline in *PAI-1^–/–^*:*SorLA^–/–^* mice treated with/without PAI-1_WT_ and in *PAI-1^–/–^*:*SorLA^+/+^* mice treated with/without PAI-1_WT_. Data are reported as mean ± SEM. *P* values are shown from 2-way ANOVA with Tukey’s multiple-comparison test (**B** and **C**), grouped 2-way ANOVA with Šidák’s test for multiple comparisons (**E**), and an unpaired *t* test (**F**). NS, not significant.

After discovering that PAI-1 binds SorLA in the injured lung, we hypothesized that, if a PAI-1–SorLA interaction is required to promote pulmonary fibrosis, then SorLA deficiency should protect against scarring. To test this hypothesis, we administered single-dose bleomycin to littermate mice either deficient (*SorLA^–/–^*), heterozygous (*SorLA^+/–^*), or WT (*SorLA^+/+^*) for SorLA expression. Lung collagen accumulation was quantified via hydroxyproline, and the pattern of scarring was assessed by histopathology. We observed that bleomycin caused a similar initial weight loss in the 3 genotypes over the first 4 days of the study (Figure 5A). Thereafter, the *SorLA^–/–^* mice rapidly recovered when compared with the other 2 groups. Relative to the *SorLA^+/+^* mice, the *SorLA*^+/–^ group exhibited a late recovery in weight. We found that the recovery of weight in the *SorLA^–/–^* and *SorLA*^+/–^ groups was associated with a statistically significant reduction in lung collagen content when compared with the *SorLA*^+/+^ mice (Figure 5B). Furthermore, we observed a SorLA gene-dose response in which lung hydroxyproline content in the *SorLA^+/–^* group was intermediate between *SorLA^+/+^* and *SorLA^–/–^* mice. Histopathology using Picrosirius red staining revealed more abundant areas of collagen staining in the bleomycin-injured *SorLA^+/+^* mice compared with the *SorLA^–/–^* group, whereas the *SorLA^+/–^* animals had an intermediate phenotype (Figure 5D). We also assessed whether SorLA expression altered PAI-1 levels in the alveolar compartment at the time of lung harvest (day 21). We found that bleomycin-induced injury increased PAI-1 levels in the *SorLA^+/+^* group compared with the control animals (Figure 5C). Bleomycin-induced injury also increased PAI-1 levels in the *SorLA^+/–^* and *SorLA^–/–^* mice, and there was no difference in the PAI-1 concentration relative to SorLA gene dose.

We next tested whether an interaction between SorLA and PAI-1 is required for PAI-1 to exert its profibrotic activity by administering bleomycin to *PAI-1^–/–^* littermate mice that were either SorLA deficient (*PAI-1^–/–^*:*SorLA^–/–^*) or had WT SorLA expression (*PAI-1^–/–^*:*SorLA^+/+^*). On day 11, subsets of each genotype were administered recombinant PAI-1_WT_ for 10 days and then lungs were analyzed for hydroxyproline content. We observed that both *PAI-1^–/–^*:*SorLA^–/–^* and *PAI-1^–/–^*:*SorLA^+/+^* mice developed statistically significantly less fibrosis than the *PAI-1^+/+^*:*SorLA^+/+^* animals. We also found that reconstitution of PAI-1 in the *PAI-1^–/–^*:*SorLA^+/+^* group resulted in an increase in lung collagen, consistent with prior experiments (Figure 5, E and F, and Figure 1; see also ref. 5). In contrast, the administration of PAI-1_WT_ to the *PAI-1^–/–^*:*SorLA^–/–^* group did not induce an increase in hydroxyproline accumulation. These results support the hypothesis that an interaction with SorLA is required for PAI-1 to express its profibrotic action.

### SorLA colocalizes with PAI-1 in cells.

**Figure 6:**
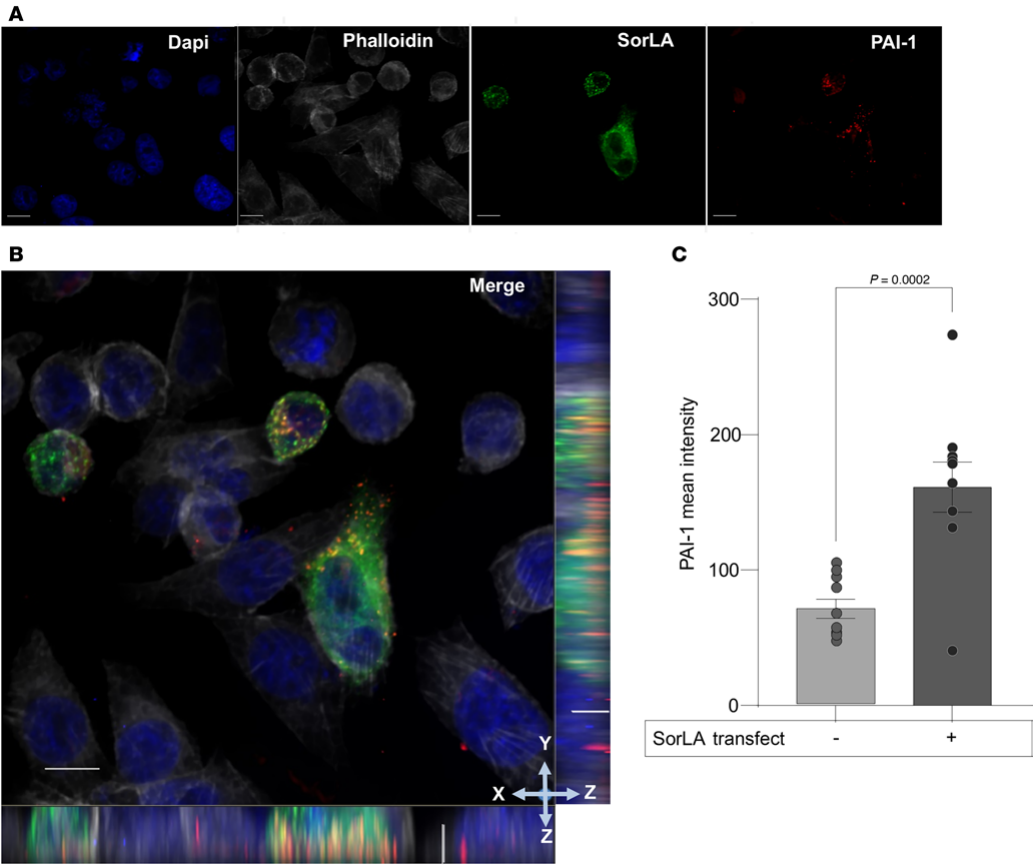
PAI-1 colocalizes with SorLA in cells. CHO cells were transfected overnight with a SorLA-GFP expression construct and then incubated with 100 nM Alexa Fluor 594–labeled PAI-1 for 1 hour. (**A**) Representative confocal images showing DAPI (blue), phalloidin (white), SorLA (green), and PAI-1 (red). (**B**) Merged image shows colocalization (yellow) of PAI-1 and SorLA in cells (*n* = 10, MOC = 0.71 ± 0.03). Rectangular panels below (*x*–*z*) and to the right (*y*–*z*) of the merged image are orthogonal views showing SorLA and PAI-1 overlap. (**C**) Mean PAI-1 in SorLA positive (+) versus SorLA negative (–) cells (*n* = 10, *P* = 0.0002). Data shown as mean ± SEM. Scale bars: 10 μm for images and 2 μm for *z*-planes.

To test whether SorLA promotes the uptake and localization of PAI-1 in cells, we transfected CHO cells, which do not express SorLA, with a SorLA-GFP construct. Twenty-four hours following transfection, cells were incubated for 1 hour with 100 nM fluorescently labeled (Alexa Fluor 594 [red]) PAI-1. Fixed cells were then analyzed by confocal microscopy. We found that SorLA-GFP and PAI-1 colocalized within cells, with a Mander’s overlap coefficient (MOC) of 0.71 ± 0.09 (Figure 6, A and B). Furthermore, SorLA expression was associated with an increase in PAI-1 uptake compared with nontransfected cells (Figure 6C).

## Discussion

PAI-1 is critical to the development of tissue fibrosis in multiple organs (2, 3, 5, 6, 23–29), and understanding its mechanism of action will shed important insight into the pathogenesis of these difficult-to-treat diseases. Early studies focused on the plasminogen activator inhibitory function and the preservation of fibrin in injured tissue as being essential to the profibrotic activity of PAI-1 (2, 30, 31). However, the demonstration that fibrin deficiency does not protect against lung fibrosis (5), and that the tPA and urokinase plasminogen activator (uPA) inhibitory activity of PAI-1 is dispensable (7), indicated that the regulation of fibrinolysis is not the primary function whereby PAI-1 exacerbates lung scarring. Furthermore, we found that PAI-1 predominantly drives pulmonary fibrosis through a mechanism that requires its VTN-binding site (7). Therefore, it was surprising to find in the present study that VTN, like fibrin, is dispensable for PAI-1 to exert its profibrotic effect. This observation indicated to us that the mutations in PAI-1 that disrupt VTN binding likely mediate interactions with other profibrotic factors. To identify additional PAI-1 interactors, we performed an unbiased proteomics analysis and discovered SorLA as the most highly enriched PAI-1 binding partner in the injured lung. This finding prompted us to investigate the role of SorLA in fibrogenesis, and we established that SorLA expression exacerbates lung fibrosis in a murine model and that SorLA is increased in human fibrotic lung tissue.

SorLA contains domains that share homology with the LDLR and the VPS10 receptor protein families. LRP1 is an LDLR family member that binds PAI-1 (32, 33). The homology between SorLA and LRP family members led Gliemann and colleagues to investigate an interaction between PAI-1 and SorLA (21). They found that LRP and SorLA possess similar affinities for PAI-1, which they suggested were mediated by the ligand binding domains on the two receptors. In the present study, we extend the understanding of the PAI-1–SorLA interaction by showing that PAI-1 also binds to the VPS10 domain. In addition, we found that mutations in PAI-1 that disrupt binding to VTN also reduce affinity for SorLA. We further discovered that the SMB domain of VTN can compete with PAI-1 binding to SorLA, indicating that the PAI-1 binding sites for VTN and SorLA overlap. However, the mutations in PAI-1_AK_ that completely disrupt VTN binding do not fully abrogate binding to SorLA, suggesting that other PAI-1 residues contribute to the SorLA interaction. We previously found that PAI-1_AK_ has significantly attenuated in vivo profibrotic activity (7), but whether this reduced profibrotic activity of PAI-1_AK_ is entirely the byproduct of impaired SorLA binding or whether these same mutations also interfere with binding to other critical factors will require additional study. Finally, both SorLA and LRP have been shown to mediate PAI-1 intracellular uptake. The internalization of PAI-1 by LRP leads to lysosomal degradation (32). With the known role of SorLA in protein uptake and sorting, we found that within CHO cells transfected with SorLA, this mosaic receptor colocalizes with PAI-1 within the cytoplasm. Based on the abundant colocalization, we speculate that SorLA internalizes PAI-1 and may circumvent targeting PAI-1 to the lysosome and instead allow trafficking to other cytoplasmic compartments, as we recently demonstrated for the uptake of Tau protein (34).

The critical role that SorLA plays in the PAI-1 profibrotic pathway is substantiated by our in vivo studies in SorLA-deficient and SorLA-heterozygous mice. Specifically, we identified a SorLA gene-dose effect in modulating the severity of lung scarring following bleomycin-induced injury. These data are remarkably congruent with what has been observed in PAI-1–deficient and PAI-1–heterozygous mice following the same insult (2). We established the importance of a PAI-1–SorLA interaction in lung fibrosis through a reconstitution experiment in *PAI-1^–/–^*:*SorLA^–/–^* mice where administering PAI-1_WT_ following lung injury did not increase fibrosis. This result contrasts with data from PAI-1 reconstitution in *PAI-1^–/–^*:*VTN^–/–^* mice (Figure 2). Finally, the increased expression of SorLA in human IPF lung tissue lends additional credence to a role for SorLA in lung fibrogenesis.

How the SorLA–PAI-1 interaction regulates fibrogenesis will require additional study, yet SorLA has been implicated in other aging-related disorders, including cardiovascular disease and Alzheimer disease (35–41). The pathogenic role of SorLA in these seemingly disparate conditions leads us to speculate about shared mechanisms. SorLA is hypothesized to impact atherosclerosis pathogenesis through internalization of cell surface receptors involved in smooth muscle cell migration, and in Alzheimer dementia, SorLA influences the intracellular trafficking and accumulation of toxic Tau and aβ protein (21, 34, 42–46). We now have evidence that SorLA can also influence PAI-1 uptake and intracellular accumulation. Regarding aging, PAI-1 levels have been linked to life-span in a Berne, Indiana Amish population and in a Klotho-deficient mouse model. PAI-1 levels have also been found to increase with age. Whether SorLA expression also changes with age will require further investigation. Beyond Alzheimer dementia and atherosclerotic disease, SorLA also facilitates insulin receptor localization and signaling, and SorLA-deficient mice are protected from high-fat feeding (47), demonstrating a phenotype that is similar to that of *PAI-1^–/–^* mice (48–51). As an extension of these pathways, it is enticing to postulate that SorLA is controlling the intracellular uptake and/or sorting of PAI-1, and this possibility is supported by our CHO cell data. How PAI-1 exerts its profibrotic function once internalized remains a matter of speculation, but several recent reports identify several intracellular functions of PAI-1 (52, 53). Alternatively, SorLA could affect how PAI-1 and LRP coordinate the signaling and recycling of cell surface receptors (54). Ultimately, understanding the pathway(s) mediated by SorLA that exacerbate fibrosis will shed light on novel therapeutic targets. Importantly, SorLA does not appear to regulate fibrogenesis by modulating PAI-1 levels, as we found no difference in BAL PAI-1 concentrations in *SorLA^+/+^*, *SorLA*^+/–^, and *SorLA^–/–^* mice on day 21 after bleomycin administration. We acknowledge, however, that our analysis of PAI-1 at a single time point may have missed an effect of SorLA on PAI-1 at an earlier stage of scarring.

To conclude, we have further elucidated the mechanism by which PAI-1 contributes to lung fibrogenesis. We confirmed the critical role of residues in PAI-1 necessary for VTN binding, but we found that VTN is dispensable in the development of fibrosis. This finding led us to discover SorLA as the most highly enriched PAI-1 binding partner in the fibrosing lung, and follow-up experiments further characterized the interaction between PAI-1 and SorLA at the cellular level. This led us to establish a critical role for SorLA in a murine model of lung fibrosis and to determine that SorLA expression is increased in human IPF lung tissue. These observations provide a deeper understanding of fibrosis pathogenesis and reveal what we believe are new therapeutic targets.

## Methods

### Sex as a biological variable.

Sex was evaluated as a biological variable by comparing the severity of lung collagen accumulation in male and female mice in both lung fibrosis models. No differences were found between sexes, and data from both sexes are combined in reported figures.

### Animals.

Mice deficient in PAI-1 (*PAI-1^–/–^*) (55) and VTN (*VTN^–/–^*) (56) were backcrossed with C57BL/6 mice for 8 or more generations. Transgenic mice expressing the human DTR (*DTR+*) under the control of the murine SPC promoter were generated in our laboratory on a C57BL/6 background (57). These mice were bred with *PAI-1^–/–^* mice (55) to generate offspring that carry a single copy of the DTR gene and are PAI-1 deficient (*DTR+*:*PAI-1^–/–^*). C57BL/6 mice (WT) and mice with a SorLA gene trap loss-of-function mutation mice (stock 007999) were purchased from The Jackson Laboratory. Heterozygous SorLA breeders were crossed, and littermates were randomized to our bleomycin model.

### PAI-1 mutant proteins and recombinant SMB.

WT and modified PAI-1 proteins were generated in collaboration with Innovative Research (58). PAI-1_RR_ contains amino acid substitutions (Thr333Arg and Ala335Arg) within the reactive center loop that do not impair VTN binding but abolish antiprotease activity (20). PAI-1_AK_ contains mutations (Arg101Ala and Gln123Lys), which abolish VTN binding but do not affect antiprotease activity (22). The SMB domain of VTN was expressed in DS2 insect cells by Strep-tag fusion (Innovative Research).

### DT administration.

Weight- and age-matched DTR-expressing mice were intraperitoneally (i.p.) injected with DT (10.0 g/kg; Sigma Chemical) once daily for 14 days as previously described (57).

### Bleomycin administration.

Weight- and age-matched mice received oropharyngeal bleomycin (2.5 U/kg in 50 μL of sterile PBS; Sigma Pharmaceuticals), as previously described (7).

### Recombinant modified PAI-1 protein administration.

*PAI-1^–/–^*:*VTN^–/–^* and *PAI-1^–/–^*:*SorLA^–/–^* transgenic mice were i.p. injected with recombinant PAI-1_RR_ or PAI-1_WT_ protein (100 μg twice daily) for 10 days as previously described (7).

### Hydroxyproline assay.

Hydroxyproline content of the lung was measured as previously described (57).

### Murine lung histology.

Left lung was inflation-fixed at 25 cmH_2_O pressure with 10% neutral-buffered formalin, removed en bloc, further fixed in 10% neutral-buffered formalin overnight, and paraffin embedded. Five-micron sections were stained using H&E or Picrosirius red methods.

### BAL.

BAL fluid was collected as previously described (7).

### BAL fluid PAI-1 concentration measurements.

Murine total and active (plasminogen activator inhibitory activity) PAI-1 concentrations were measured in BAL fluid using a microsphere-based ELISA (Luminex) as previously described (7).

### MS proteomic analysis.

Bleomycin-injured lungs from *PAI-1^–/–^* mice were harvested on day 10, snap frozen, pulverized into a fine powder using a cyrogrinder (Black and Decker), and resuspended in 2.5 mL RIPA buffer (150 mM NaCl, 50 mM Tris, pH 8.0, 1% Triton X-100, 0.5% sodium deoxycholate, 0.1% SDS) with 25 μL protease inhibitor cocktail (Protease Inhibitor Cocktail Set III, EDTA-Free from Calbiochem). Lung homogenates were centrifuged and the supernatants aliquoted. Biotinylated PAI-1_WT_ (125 μg), containing 4 stabilizing substitutions (20), was bound to 125 μL of streptavidin-Sepharose beads (GE Healthcare). PAI-1–streptavidin-Sepharose beads were added to 500 μL of lung supernatant and incubated at 37°C for 1 hour with gentle agitation. Control lung homogenates were mixed with unbound streptavidin-Sepharose beads. The control and PAI-1–bound streptavidin-Sepharose beads were washed, resuspended in PBS with or without 150 μL of 12.5 mM DSP reversible cross linker (Thermo Fisher Scientific), and incubated 30 minutes at room temperature. Control and PAI-1–bound streptavidin-Sepharose beads were then washed 3 times with 1.2 mL of 2 M urea (in 1× Tris-buffered saline [TBS]), resuspended in PBS, and submitted to the MS core for analysis.

In the MS core, the beads were resuspended in 50 μL of 0.1 M ammonium bicarbonate buffer (pH 8). Cysteines were reduced by adding 50 μL of 10 mM DTT and incubating at 45°C for 30 minutes. Samples were cooled to room temperature and alkylation of cysteines was achieved by incubating with 65 mM 2-chloroacetamide, under darkness, for 30 minutes at room temperature. An overnight digestion with 1 μg sequencing grade, modified trypsin was carried out at 37°C with constant shaking in a Thermomixer. Digestion was stopped by acidification and peptides were desalted using SepPak C18 cartridges using manufacturer’s protocol (Waters). Samples were completely dried using vacufuge. Resulting peptides were dissolved in 9 μL of 0.1% formic acid and 2% acetonitrile solution and 2 μL of the peptide solution was resolved on a nano-capillary reverse-phase column (Acclaim PepMap C18, 2 μm, 50 cm, Thermo Fisher Scientific) using a 0.1% formic acid and 2% acetonitrile (buffer A) and 0.1% formic acid and 95% acetonitrile (buffer B) gradient at 300 nL/min over a period of 180 minutes (2%–25% buffer B in 110 minutes, 25%–40% in 20 minutes, 40%–90% in 5 minutes, followed by holding at 90% buffer B for 10 minutes and re-equilibration with buffer A for 30 minutes). Eluent was directly introduced into a Q Exactive HF mass spectrometer (Thermo Fisher Scientific) using an EasySpray source. MS1 scans were acquired at 60K resolution (automatic gain control target [AGC] = 3 × 10^6^; max injection time [IT] = 50 ms). Data-dependent collision induced dissociation MS/MS spectra were acquired using the Top Speed method (3 seconds) following each MS1 scan (normalized collision energy [NCE] ~28%; 15K resolution; AGC target 1 × 10^5^; max IT 45 ms).

Proteins were identified by searching the MS/MS data against UniProt mouse protein database (17,180 entries) using Proteome Discoverer (v2.1, Thermo Fisher Scientific). Search parameters included MS1 mass tolerance of 10 ppm and fragment tolerance of 0.2 Da (2 missed cleavages were allowed), carbamidimethylation of cysteine was considered fixed modification, and oxidation of methionine and deamidation of asparagine and glutamine were considered as potential modifications. False discovery rate (FDR) was determined using Percolator (ThermoScientific) and proteins/peptides with an FDR of 1% or less were retained for further analysis.

Spectral counts for each identified protein are represented as the log_2_FC between PAI-1 and control samples with (+) and without (–) crosslinker. The combined FC score for each protein was calculated as a geometric mean of the 2 individual FC scores. Results are reported as FC = (spectral counts in PAI-1 samples/spectral counts in control samples), which was calculated for each cross-linking condition and overall. For control samples where no spectral counts were detected, an arbitrary value of 0.2 was imputed to prevent dividing by zero.

The MS proteomics data have been deposited to the ProteomeXchange Consortium via the PRIDE (URL https://proteomecentral.proteomexchange.org/) partner repository with the dataset identifier PXD054196.

### Human lung tissue.

Lung tissue from patients with IPF were obtained from explanted organs obtained at the time of transplant. The diagnosis of IPF was established by multidisciplinary clinical consensus prior to transplant, and all explanted lung tissue was later confirmed by pathology to demonstrate a histopathologic diagnosis of usual interstitial pneumonia, the pathologic correlate of IPF.

### Western blotting.

Protein concentrations were measured with a Pierce BCA Protein Assay Kit (Thermo Fisher Scientific, 23227). Protein lysates were separated in 4%–15% Tris-glycine gradient gels (Bio-Rad, 4561084) and transferred overnight at 4°C to nitrocellulose membranes (Cytiva, 10600008). Membranes were blocked at room temperature with Intercept Blocking Buffer (Li-COR, 927-60001) and incubated overnight at 4°C with primary antibodies. Membranes were then washed with TBS/0.1% Tween 20, incubated with appropriate secondary antibodies for 1 hour at room temperature, washed, and imaged on an Odyssey CLx (Li-COR). Primary antibodies against vinculin (2 μg/mL; Santa Cruz Biotechnology, mouse monoclonal, sc-25336), SorLA (2 μg/mL; Abcam, rabbit monoclonal, ab190684), or αSMA (1:100; Invitrogen, mouse monoclonal, MA5-11547) was used. Donkey anti-rabbit-800 (1:20,000; Li-COR, 926-32213) or goat anti-mouse-700 (1:20,000; Li-COR, 926-68070) was used for the secondary antibody.

### SorLA expression in human lungs.

Human lung tissue from patients with IPF and healthy controls was homogenized in ice-cold RIPA buffer supplemented with protease inhibitor (defined previously), and supernatants were collected following centrifugation (10,000*g* for 20 minutes, twice). Protein (30 μg) was separated by SDS-PAGE and analyzed for SorLA, αSMA, and vinculin expression by Western blotting. Active PAI-1 expression was measured by ELISA.

### Binding of PAI-1 to SorLA in human lung tissue.

Human IPF lung tissue was homogenized in ice-cold binding buffer (20 mM Tris, pH 8.0, 140 mM NaCl, 10 mM CaCl_2_, 10% glycerol, 1% NP-40) supplemented with protease inhibitor and supernatants collected following centrifugation (11,000*g* for 10 minutes at 4°C, twice). Fifty microliters of Streptavidin Mag Sepharose beads (Cytiva, 28-9857-38) were washed with binding buffer and incubated with or without 10 μg of biotinylated PAI-1_WT_ (~67% of bead capacity) for 30 minutes at room temperature with gentle agitation. Beads were washed (50 mM Tris, pH 7.4, 150 mM NaCl), and then incubated with 200 μg of lung lysates for 2 hours at room temperature with gentle agitation. Beads were washed to remove nonspecific binding and boiled in 2× Laemmli sample buffer (Bio-Rad, 1610737) supplemented with 2-mercaptoethanol (Bio-Rad, 1610710) for 5 minutes at 100°C to elute protein. Eluted protein and 50 μg of original sample (input) were analyzed by Western blotting.

### PAI-1 and SorLA staining in normal and IPF lung tissue.

Duplex immunofluorescence was performed on a Ventana Discovery Ultra stainer. Slides were dewaxed, rehydrated, and subjected to heat-induced epitope retrieval on board the stainer. Slides were then subjected to sequential incubation with anti–PAI-1 (1:300; rabbit polyclonal, Innovative Research, ASHPAI-GF-HT) for 1 hour, goat anti–rabbit IgG conjugated to HRP for 16 minutes (Ventana, 760-4311), and developed with Akoya Opal 520 (1:150; Akoya Biosciences, NC1601877) for 12 minutes. After an additional round of heat-induced epitope retrieval to remove the PAI-1 primary antibody–secondary antibody complex, the slides were stained with anti-SorLA (1:1,000; rabbit monoclonal, Abcam, ab190684) for 28 minutes, goat anti–rabbit IgG conjugated to HRP, and developed with Akoya Opal 690 (1:150; Akoya, NC1605064) for 12 minutes. Slides were then counterstained and coverslipped using Prolong Gold containing DAPI (Invitrogen, P36931). After drying, slides were imaged on an Akoya Polaris scanner with the following exposure times (DAPI, 4.5 ms, Opal 520, 6.3 ms, and Opal 690, 28.3 ms). Serial sections were stained with H&E.

### SPR.

Purified full-length SorLA or its VSP10 domain (Bio-Techne) were immobilized on a CM5 sensor chip surface to the levels of 5,000 and 2,500 response units respectively, using a working solution of 20 μg/mL of protein in 10 mM sodium acetate, pH 4. An additional flow cell was activated and blocked with 1.0 M ethanolamine without protein to act as a control surface. Unless otherwise stated, binding experiments were performed in HBS-P buffer (0.01 M HEPES, 0.15 M NaCl, 0.005% surfactant P, 1.0 mM CaCl_2_, pH 7.4). Experiments were performed either on a BIAcore 3000 instrument or a Biacore 8K instrument, using a flow rate of 20 μL/min at 25°C. Sensor chip surfaces were regenerated by 30-second injections of 10 mM glycine/20 mM EDTA/0.5 M NaCl pH 4 at a flow rate of 30 μL/min. Equilibrium binding data for PAI-1_WT_ and PAI-1_AK_ were determined by fitting the association rates to a pseudo–first-order process to obtain *R_eq_*. *R_eq_* was then plotted against total ligand concentration, and the data were normalized to *R_eq_*/*R_max_* to account for different amounts of protein coupled to the surface. For the inhibition of PAI-1_WT_ binding to full-length SorLA by the SMB domain of VTN, 250 nM PAI-1_WT_ was preincubated with increasing concentrations of SMB prior to SPR analysis. The data were fit to a binding isotherm using nonlinear regression analysis in GraphPad Prism 10 software using the following equation: *y* = *B_max_* × *L*/(*K_D_* + *L*), where *B_max_* is the *R_eq_* value at saturation, *L* is the free ligand concentration, and *K_D_* is the equilibrium binding constant.

### SorLA transfection and PAI-1 colocalization.

CHO cells were seeded on Lab-Tek II chamber slides (Thermo Fisher Scientific, 154461) coated with 0.1 mg/mL of Type I Bovine Collagen (Advanced BioMatrix, 5005-100ML) the night before transfection. Cells were transfected with 1 μg of SorLA-GFP expression construct (gift from Olav Andersen, Aarhus University, Aarhus, Denmark) using Lipofectamine 3000 Transfection Reagent (Invitrogen, L3000001) according to the manufacturer’s protocol. Twenty-four hours following transfection, cells were incubated for 1 hour with 100 nM Alexa Fluor 594–labeled PAI-1 (Innovative Research, IHUPAI1ARAF5941MG). Cells were fixed using 10% buffered formalin (Fisher Chemical, SF100-4) and stained with DAPI (1:1000; Invitrogen, 62247) and phalloidin (1:400; Invitrogen, A22287) according to the manufacturer’s protocol. *Z*-stack images (0.25-μm steps) were captured using a 60× oil immersion objective on a Nikon Eclipse Ti spinning disk confocal microscope. Standardized background subtraction was performed on all images in NIS-Elements AR imaging software. Colocalization was assessed by MOC calculated for GFP (SorLA) and Texas red (PAI-1) channels in NIS-Elements. MOC measures the co-occurrence of pixels, where values range from 0 to 1 (0 indicates no overlapping pixels and 1 indicates absolute colocalization). To determine whether there was a difference in the amount of cell-associated PAI-1, the mean PAI-1 signal intensity was analyzed in cells with and without SorLA-GFP transfection using ImageJ (NIH).

### Statistics.

Data are presented as mean ± standard error of the mean (SEM). For statistical analysis, GraphPad Prism software was used. In any experiment with only 2 groups, a 2-tailed *t* test was used. For experiments with more than 2 groups, a 2-way ANOVA was used with Tukey’s post hoc test for multiple comparisons. Outliers in all data sets were identified using the Prism ROUT test. A *P* value of less than 0.05 was considered significant.

### Study approval.

Animal experiments were reviewed and approved by the University of Michigan Committee on the Use and Care of Animals (UCUCA, Ann Arbor, Michigan, USA). Human lung tissue were obtained from patients who provided informed consent, and the study was approved by the University of Michigan Institutional Review Board (HUM00105694, Ann Arbor, Michigan, USA). Nonfibrotic lungs that were rejected for transplantation were obtained from healthy controls donated by Gift of Life, Michigan.

### Data availability.

For original data, please contact the corresponding author. The mass spectrometry proteomics data have been deposited to the ProteomeXchange Consortium via the PRIDE [1] partner repository with the dataset identifier PXD054196. Values for all data points in graphs are reported in the Supporting Data Values file.

## Author contributions

THS and JJO co-authored a first draft of the manuscript. Multiple subsequent revisions including the incorporation of new data, and conclusions were made by THS to generate the final draft. THS (with DAL) conceived of, designed, supervised and interpreted all experiments for the manuscript. JJO assisted in planning experiments, interpreting data, and developing figures. LL performed PAI-1–SorLA binding experiments in IPF lung tissue, the SotLA-mediated PAI-1 uptake in CHO cells, and contributed to manuscript preparation. VB and AN assisted in designing the proteomics experiment and interpreting of the MS data. NS performed all in vivo experiments. AQV performed the ex vivo MS experiments. SSG captured mouse histology images and aided in figure preparation. JCH assisted with data interpretation and manuscript preparation. MW performed Western blotting on human lung tissue and assays for PAI-1 concentrations and activity. DT performed SorLA pull-down experiments with PAI-1–labeled streptavidin beads. MM and DKS designed, performed, interpreted, and helped report the SPR studies. KKK provided baseline hydroxyproline data from uninjured VTN^null^ mice. SKH provided explanted human lung tissue samples from patients with advanced fibrosis and tissue from healthy controls (rejected at time of transplantation). DAL constructed the PAI-1 mutant proteins, and he assisted in the planning of the experiments, data interpretation, and manuscript preparation. THS generated the hypothesis, planned the experiments, interpreted the data, and authored the manuscript.

## Supplementary Material

Supplemental data

Unedited blot and gel images

Supporting data values

## Figures and Tables

**Table 1:**
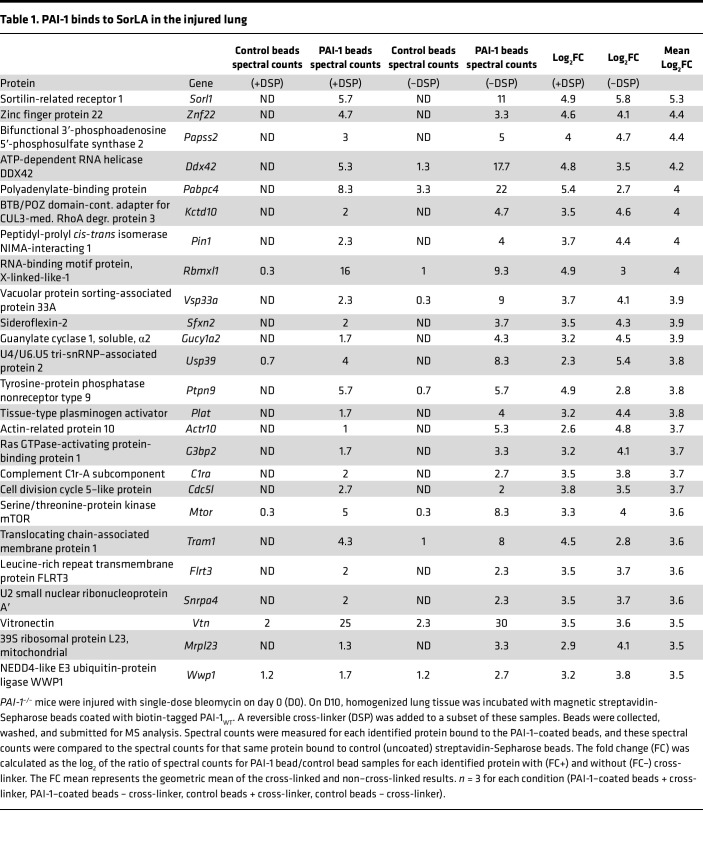
PAI-1 binds to SorLA in the injured lung

**Table 2:**
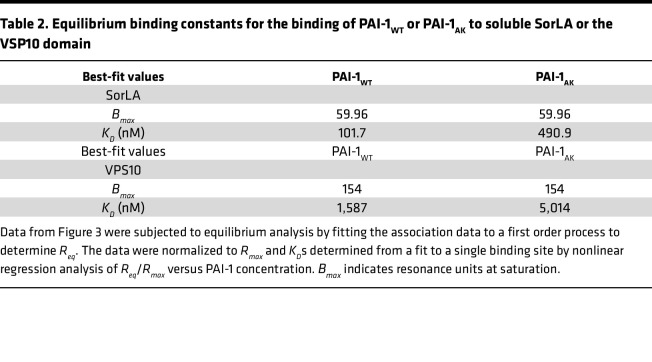
Equilibrium binding constants for the binding of PAI-1_WT_ or PAI-1_AK_ to soluble SorLA or the VSP10 domain

## Version 1 04/29/2025

In-Press Preview

## Version 2 06/09/2025

Electronic publication
