# Supplementary material for: PAI-1 interaction with sortilin-related receptor 1 is required for lung fibrosis
Source: JCI Insight. 2025 Apr 29;10(11):e186131. doi: 10.1172/jci.insight.186131 (PMC12220977; doi:10.1172/jci.insight.186131)
Supplement: Supplemental data [file jciinsight-10-186131-s230.pdf]

Supplemental Figure 1

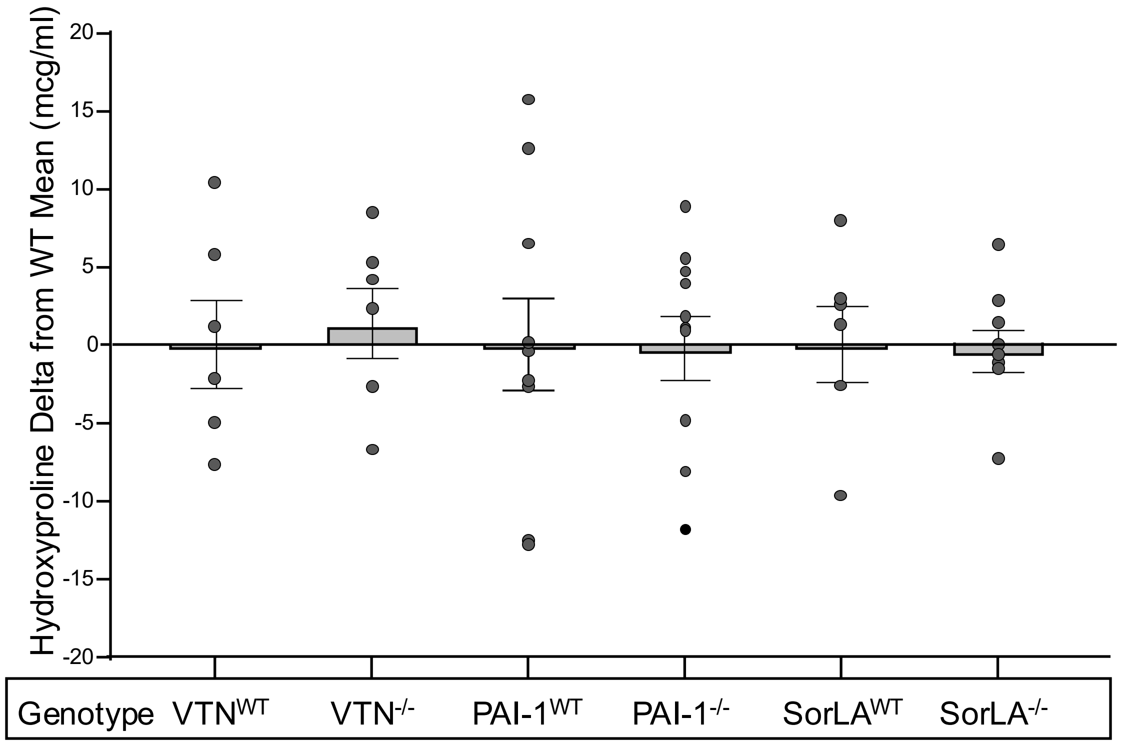

Supplemental Figure 1. Baseline lung collagen content in VTN^{Null}, PAI-1^{Null}, and $SorlA^{Null}$ mice. Uninjured WT mice were compared to uninjured **(A)** VTN^{Null}, **(B)** PAI-1^{Null}, and **(C)** $SorlA^{Null}$ mice with respect to their baseline lung hydroxyproline content. Data are reported as the mean difference in lung hydroxyproline compared to the control (WT) groups ± SEM. P values are shown for comparisons performed using a parametric two tailed t-test. NS = non-significant.

Supplemental Figure 2

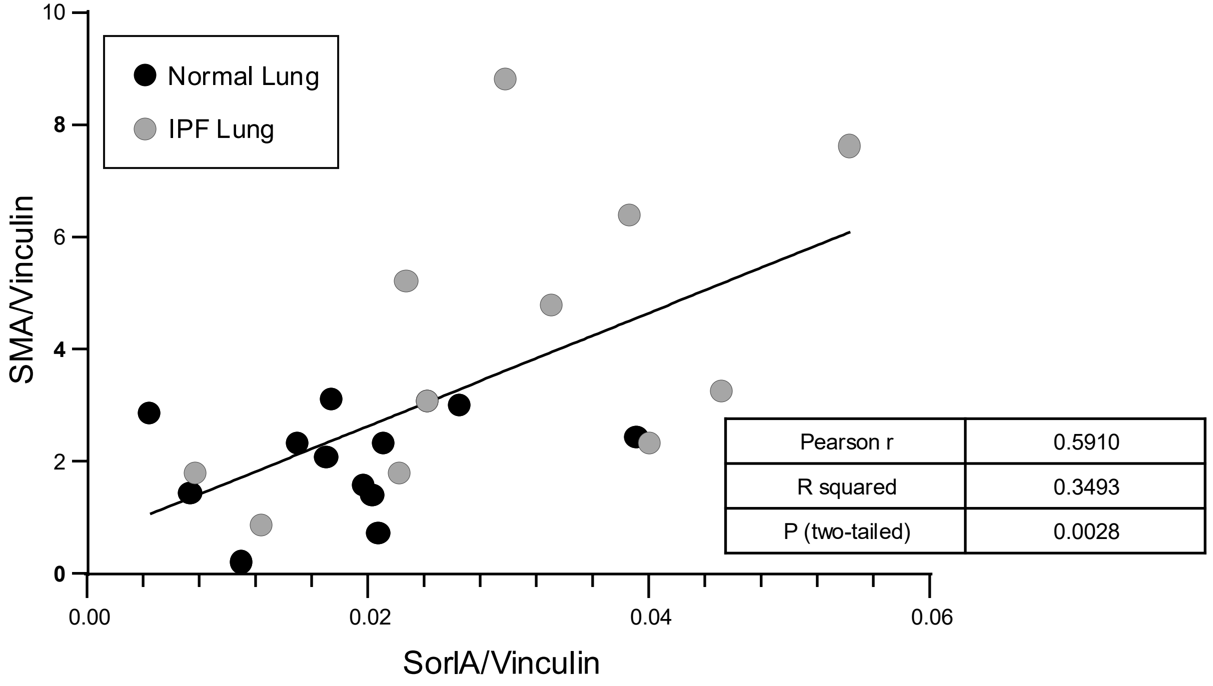

Supplemental Figure 2. Correlation between SorlA protein and SMA protein levels in normal and IPF lung tissue. Individual values for SorlA and SMA protein levels (assessed by densitometry and normalized to vinculin) from 11 normal and 11 IPF lung tissue samples were plotted and analyzed by a Pierson Correlation Coefficient.
