# Supplementary figures and images for: PAI-1 interaction with sortilin-related receptor 1 is required for lung fibrosis
Source: JCI Insight. 2025 Apr 29;10(11):e186131. doi: 10.1172/jci.insight.186131 (PMC12220977; doi:10.1172/jci.insight.186131)

# Figure 4A

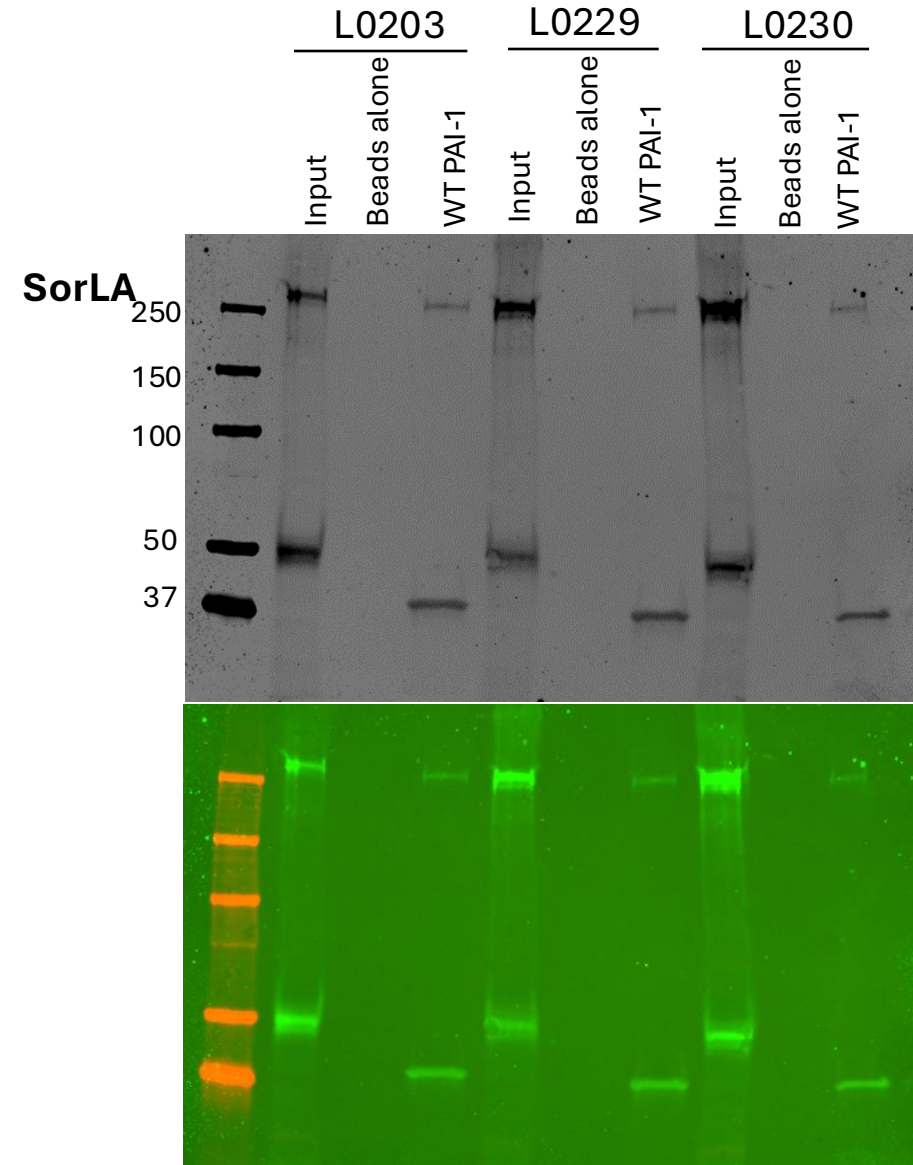

# Figure 4B

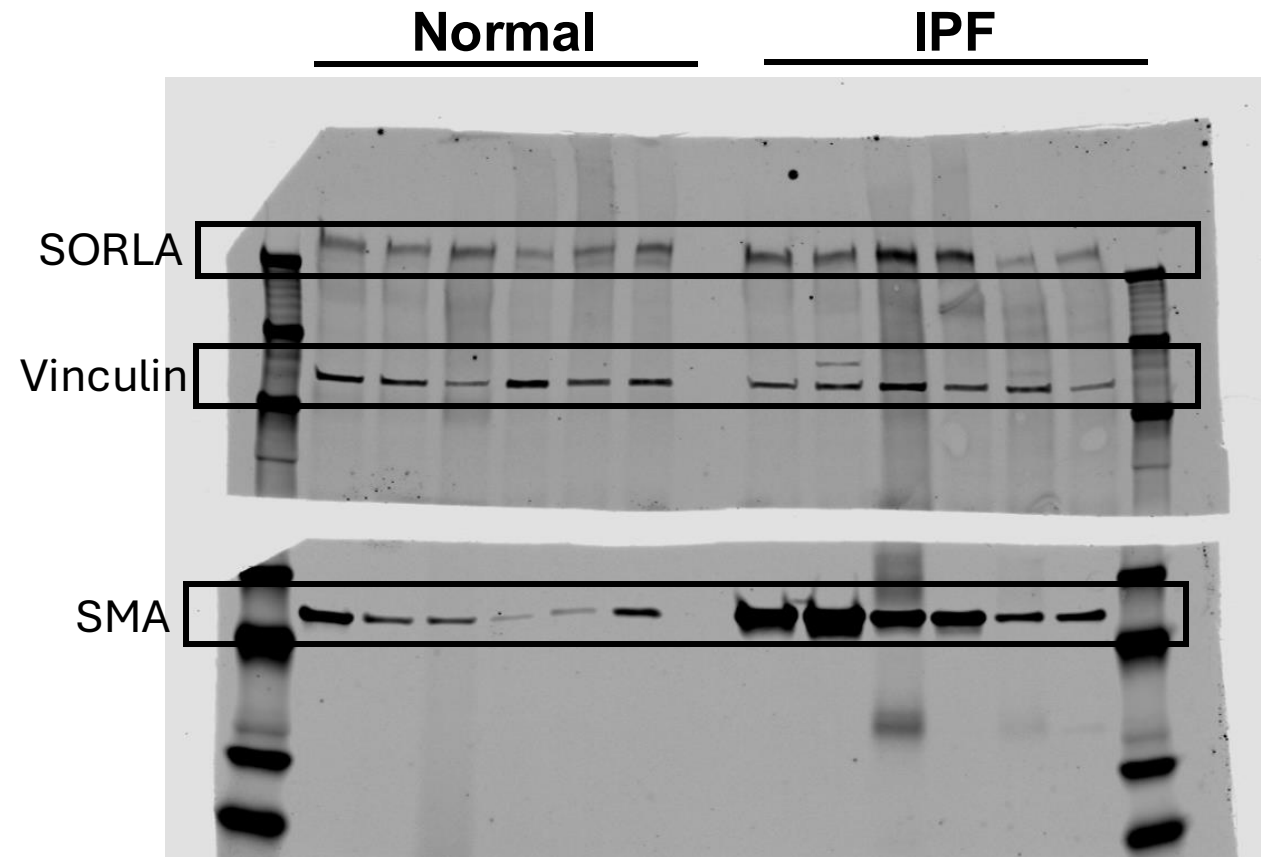

Supplement: Unedited blot and gel images [file jciinsight-10-186131-s231.pdf]
